# ﻿Divergent karyotypes in five genera of the African endemic fish family Distichodontidae (Cithariniformes, Osteichthyes)

**DOI:** 10.3897/compcytogen.17.107744

**Published:** 2023-11-02

**Authors:** Sergey A. Simanovsky, Dmitry A. Medvedev, Fekadu Tefera, Alexander S. Golubtsov

**Affiliations:** 1 Severtsov Institute of Ecology and Evolution, Russian Academy of Sciences, Leninsky Prospect 33, Moscow, 119071 Russia Severtsov Institute of Ecology and Evolution, Russian Academy of Sciences Moscow Russia; 2 National Fishery and Aquatic Life Research Center, Ethiopian Institute of Agricultural Research, Sebeta, P.O. Box 64, Ethiopia National Fishery and Aquatic Life Research Center, Ethiopian Institute of Agricultural Research Sebeta Ethiopia

**Keywords:** Africa, chromosomes, *Distichodus*, *Ichthyborus*, karyotype evolution, *Nannaethiops*, *Nannocharax*, *Neolebias*

## Abstract

The African family Distichodontidae comprises 109 species in 16 genera. Up-to-date cytogenetic information was available for the only distichodontid species *Distichodusaffinis* Günther, 1873. Here we report chromosome number and morphology in: *Distichodusengycephalus* Günther, 1864 (2n = 52, FN = 104), *Ichthyborusbesse* (Joannis, 1835) (2n = 46, FN = 92), *Nannocharaxniloticus* (Joannis, 1835) (2n = 54, FN = 106) and three taxa, *Nannaethiopsbleheri* Géry et Zarske, 2003, *Nannaethiops* sp., and *Neolebiasunifasciatus* Steindachner, 1894, that exhibit the same karyotypes (2n = 50, FN = 98). To confirm the *Nannaethiops* Günther, 1872 and *Neolebias* Steindachner, 1894 species identification, mt-DNA sequences of the two markers (*COI* and *16S rRNA*) were obtained from karyotyped specimens and compared with the relevant sequences accessible from GenBank. The great prevalence of biarmed chromosomes (the karyotypes of most species contain exclusively biarmed chromosomes) is a distinctive characteristic of Distichodontidae and Cithariniformes as a whole.

## ﻿Introduction

Until recently the two Afrotropical families, Citharinidae and Distichodontidae, were considered as belonging to characins, the order Characiformes, classified into two suborders: Citharinoidei with 117 species in two Afrotropical families and Characoidei with more than 2000 species in two Afrotropical and 20 Neotropical families (Nelson et al. 2016; Froese and Pauly 2023). Recently, however, sister group relationships between Characoidei and catfishes, the order Siluriformes, has been inferred from the molecular data (Melo et al. 2022). Therefore, Cithariniformes along with Characiformes (containing former Characoidei only) and Siluriformes should be recognized as distinct orders (Dornburg and Near 2021).

While Citharinidae include eight species in three genera, Distichodontidae are more species rich including 109 species in 16 genera (Eschmeyer et al. 2023, Froese and Pauly 2023). The molecular phylogeny of Citharinoidei is well established: there is the distinct family Citharinidae and six clades within the family Distichodontidae (Arroyave et al. 2013; Lavoué et al. 2017). Two representatives of the former family – *Citharinuscitharus* (Geoffroy St. Hilaire, 1809) and *C.latus* Müller et Troschel, 1844 – and the only representative of the latter family – *Distichodusaffinis* Günther, 1873 – were studied cytogenetically (Rab et al. 1998; Simanovsky et al. 2022). All three studied species have exclusively biarmed karyotypes with 2n = 40, 44 and 48 (for *C.citharus*, *C.latus and D.affinis*, respectively). Six distichodontid species from the five genera – *Distichodus* Müller et Troschel, 1844; *Ichthyborus* Günther, 1864; *Nannocharax* Günther, 1867; and *Nannaethiops* Günther, 1872 and *Neolebias* Steindachner, 1894 – involved in this study represent the four out of six clades identified by molecular methods within the family (Arroyave et al. 2013; Lavoué et al. 2017).

The present study is aimed at an estimation of the divergence of the karyotype structure (the number and morphology of chromosomes) between and within the phylogenetically distant lineages of the family Distichodontidae. The concordance between differences in karyotype structure and the molecular phylogenies elaborated for the family Distichodontidae by the previous researchers is considered.

## ﻿Material and methods

### ﻿Sample acquisition and characteristics

Ethiopian material was obtained from tributaries of the Sobat River, a tributary of the White Nile, in southwestern Ethiopia (Table 1). Fish were collected by the Joint Ethio-Russian Biological Expedition (JERBE) with the permissions of the National Fisheries and Aquatic Life Research Center under the Ethiopian Institute of Agricultural Research (EIAR) and the Ethiopian Ministry of Science and Technology. Two individuals – male and female – of *Nannaethiopsbleheri* Géry et Zarske, 2003, collected from the roadside ditch in the interfluve of the Alvero and Gilo rivers (between towns of Abobo and Funido, 7°45.307'N, 34°15.639'E) were karyotyped. The rest of karyotyped Ethiopian material was obtained from the two localities: (1) Alvero River just downstream of the Abobo Dam (7°52.503'N, 34°29.960'E) and (2) Baro River at the City of Gambela (8°14.878'N, 34°34.044'E). Two males and a female of *Distichodusengycephalus* Günther, 1864, as well as a female of *Ichthyborusbesse* (Joannis, 1835), were collected at locality 1. Two males of *I.besse* and a female of *Nannocharaxniloticus* (Joannis, 1835), were collected at locality 2.

**Table 1.** Species, fish standard length (SL), numbers of individuals (N) and metaphases (N_mt_) studied, and collection site. UD – undetermined sex.

Species	SL, mm	N	N_mt_	Collection site
*Distichodusengycephalus*	149–163	3 (1♀, 2♂)	30	Alvero River
*Ichthyborusbesse*	110	1 (1♀)	25	
	103–118	2 (2♂)	20	Baro River
*Nannocharaxniloticus*	51	1 (1♀)	10	
*Nannaethiopsbleheri*	19–23	2 (1♀, 1♂)	20	Interfluve of the Alvero and Gilo rivers
*Nannaethiops* sp.	23–26	4 (1♀, 2♂, 1UD)	40	West Africa (fish store)
*Neolebiasunifasciatus*	25–31	7 (5♀, 2♂)	81	

Four specimens (a female, two males and one unsexed) of an unidentified species representing the genus *Nannaethiops* and seven specimens (five females and two males) of *Neolebiasunifasciatus* Steindachner, 1894 were purchased from the Nigerian aquarium fish dealers through the mediation of the company Aqua Logo Engineering (https://www.aqualogo-engineering.ru).

After colchicine treatment, fish were euthanized with an overdose of tricaine methanesulfonate (MS-222), identified, measured with an accuracy of 1 mm, dissected for gonad examination and tissue sampling, and preserved in 10% formaldehyde or 70% ethanol. Species identification was done based on morphological characters (Gosse and Coenen 1990; Golubtsov et al. 1995). The experiments were carried out in accordance with the rules of the Severtsov Institute of Ecology and Evolution (IEE) and approved by IEE’s Ethics Committee. Vouchers are deposited at the Severtsov Institute of Ecology and Evolution (Moscow), under provisional labels of JERBE.

### ﻿DNA extraction, PCR amplification, and sequencing

In order to clarify the phylogenetic position of *Nannaethiops* and *Neolebias* specimens, two genetic markers – *Cytochrome oxidase subunit I (COI)* and *16S ribosomal RNA (16S rRNA)* – were studied in 13 karyotyped fish and one additional specimen of *N.bleheri* from an another location in Ethiopia (Suppl. material 1: table S1). We extracted total genomic DNA from the ethanol-preserved tissues using the DiatomDNA Prep 100 (Izogen, Moscow) extraction kit. The PCR mixture contained 5 pmol of each primer and the precast PCR mixture from DIALAT Ltd (Russia). The primers used for *COI* amplification were designed by Ward et al. (2005): FishF1-5′TCAACCAACCACAAAGACATTGGCAC3′ and FishR1-5′TAGACTTCTGGGTGGCCAAAGAATCA3′. The PCR cycle profiles were as follows: 5 min initial denaturation at 94 °C, followed by 35 cycles of 1 min at 94 °C, annealing for 45 sec at 55 °C, extension for 1 min at 72 °C; final extension for 7 min at 72 °C. The primers 8f-5′AGAGTTTGATCCTGGCTCAG3′ (Edwards et al. 1989) and 1492r-5′GGTTACCTTGTTACGACTT3′ (Stackebrandt and Liesack 1993) were employed for the *16S rRNA* amplification. The PCR cycle profiles were as follow: 3 min initial denaturation at 94 °C, followed by 30 cycles of 30 sec at 94 °C, annealing for 30 s at 50 °C, extension for 30 sec at 72 °C; final extension for 7 min at 72 °C. PCR products were visualized by electrophoresis in 1.5% agarose gel in TBE buffer with addition of ethidiumbromide. DNA sequencing was performed using an Applied Biosystems 3500 genetic analyzer. All new DNA sequencies were deposited in GeneBank (Suppl. material 1: table S1).

### ﻿Sequence alignment and phylogenetic reconstruction

Preprocessing and alignment of the obtained sequences was carried out using SeqMan Pro 7.1.0 and BioEdit 5.0.9. For phylogenetic reconstruction all sequences of the two markers (*COI* and *16S rRNA*) available in GenBank for *Nannoethiops* and *Neolebias* specimens were used. These sequences are listed below. The distichodontid species *Belonophagohutsebouti* Giltay, 1929, *Distichodusnefasch* (Bonnaterre, 1788) and *D.sexfasciatus* Boulenger, 1897, as well as citharinid *Citharinuscitharus* (Geoffroy Saint-Hilaire, 1809), were selected as outgroups. The GenBank accession numbers for outgroups are given in Suppl. material 1: table S1.

**Figure 1.**
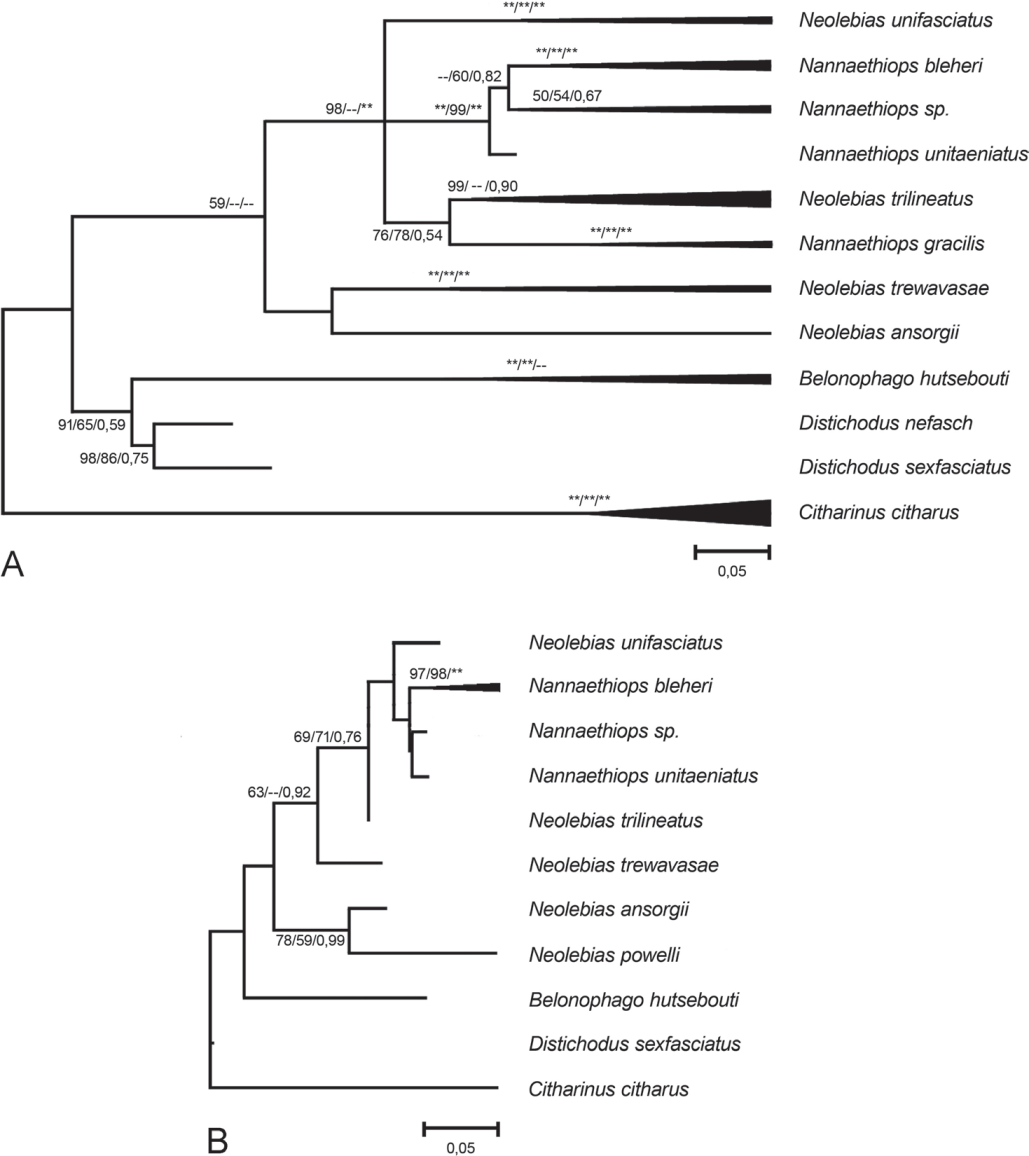
Maximum Likelihood (ML) trees with compressed subtrees based on (**A**) 615-bp *COI* fragment and (**B**) 387-bp *16S rRNA* fragment. Length of branches is proportional to the genetic distances between haplotypes; bootstrap support (Felsenstein, 1985) is indicated next to the branching nodes and calculated with ML/Maximum Parsimony/Bayesian Inference methods from 1000 replicas (“*” - bootstrap support is equal to 100% or 1, “-- ” or not specified - bootstrap support is less than 50%).

Comparative material included the GenBank sequences of six species representing the genera *Nannoethiops* and *Neolebias* for *CO1* and seven such species for *16S rRNA* (Fig. 1, Suppl. material 1: table S1). For *CO1*, these were *Nannaethiopsbleheri* from Ethiopia (the GenBank accession number KF541848, Arroyave et al. 2013), *Nannaethiopsgracilis* (Matthes, 1964) (KF541851, KF541852, Arroyave et al. 2013), *Nannaethiopsunitaeniatus* Günther, 1872 (KF541849, KF541850, Arroyave et al. 2013), *Neolebiasansorgii* Boulenger, 1912 (KF541858, KF541859, KF541860, Arroyave et al. 2013; HM418212, HM418213, Sonet et al. 2019), *Neolebiastrewavasae* Poll et Gosse, 1963 (KF541853, KF541857, Arroyave et al. 2013) and *Neolebiastrilineatus* Boulenger, 1899 (KF541854, KF541855, KF541856, Arroyave et al. 2013; KT193336, Decru et al. 2016; HM418214, HM418215, MK074510, MK074511, Sonet et al. 2019), all from West Africa. For *16S rRNA*, these were *Nannaethiopsbleheri* from Ethiopia (JX985104, Lavoué et al. 2017), *Nannaethiopsunitaeniatus* (JX985105, Lavoué et al. 2017), *Neolebiasansorgii* (AY788058, Calcagnotto et al., 2005; JX985107, Lavoué et al. 2017), *Neolebiaspowelli* Teugels et Roberts, 1990 (AY788061, Calcagnotto et al. 2005), *Neolebiastrewavasae* (JX985132, Lavoue et al. 2017), *Neolebiastrilineatus* (AY788063, Calcagnotto et al. 2005) and *Neolebiasunifasciatus* Steindachner, 1894 (JX985103, Lavoué et al. 2017), all from West Africa.

For phylogenetic reconstruction, we used Maximum Likelihood (ML), Maximum Parsimony (MP) (Nei and Kumar 2000) and Bayesian Inference (BI) methods. For ML, the chosen models of molecular evolution were as follows: Hasegawa-Kishino-Yano (HKY +G+I; parameter +G = 1.77; +I = 0.6) (Hasegawa et al. 1985) for *COI* and Tamura-Nei (TN93+G; parameter +G = 0.13) (Tamura and Nei 1993) for *16S rRNA*. For ML and MP, the bootstrap support for branch nodes was calculated with 1,000 replicates (Felsenstein 1985). Genetic distances and other parameters for phylogenetic ML and MP analysis were calculated using the MEGA X software package (Kumar et al. 2018). The nucleotide substitution model for BI was selected by means of the Bayesian Information Criterion (BIC) as implemented in jModelTest (Posada 2008). BI was carried out in MrBayes version 3.1.2 (Huelsenbeck and Ronquist 2001; Ronquist and Huelsenbeck 2003) and implemented using Markov Chain Monte Carlo algorithm for 10,000 generations with a sampling period of 1,000 generations.

### ﻿Cytogenetic analysis

Before preparation, fish were treated intraperitoneally with 0.1% colchicine (0.01 ml / 1 g of their weight; for Ethiopian material, under field conditions) or 0.025% colchicine (0.01 ml / 1 g of their weight; for Nigerian material, under laboratory conditions) for 3–5 hours. After euthanasia, chromosome preparations were obtained from kidney tissue following Kligerman and Bloom (1977) for Ethiopian and Nigerian material or from kidney, spleen, intestine and liver following Bertollo et al. (2015) for Nigerian material with some modifications for both protocols, as described in Simanovsky et al. (2022). The chromosome spreads were stained conventionally with 4% Giemsa solution in a phosphate buffer solution at pH 6.8 for 8 min and then analysed using an Axioplan 2 Imaging microscope (Carl Zeiss, Germany) equipped with a CV-M4^+^CL camera (JAI, Japan) and Ikaros software (MetaSystems, Germany). Final images were processed using Photoshop software (Adobe, USA). Karyotypes were arranged according to the centromere position following the nomenclature of Levan et al. (1964), but modified as metacentric (m), submetacentric (sm) and subtelocentric/acrocentric (st/a). Chromosome pairs were arranged according to their size in each chromosome category. To determine the chromosomal arm number per karyotype (fundamental number, FN), metacentrics and submetacentrics were considered as biarmed, and subtelocentrics/acrocentrics as monoarmed. The total numbers of complete metaphases studied for each species is presented in Table 1.

## ﻿Results and discussion

### ﻿Molecular phylogenetic analysis

An analysis of 615 bp of the mitochondrial *CO1* in 13 individuals representing the genera *Nannoethiops* and *Neolebias* and 387 bp of the mitochondrial *16S rRNA* in seven individuals representing the same genera included the Ethiopian samples of *Nannaethiopsbleheri*, as well as the West African samples (from the Nigerian aquarium fish dealers) of the genera *Nannoethiops* and *Neolebias*. The alignment used for phylogenetic reconstructions included 47 *CO1* sequences and 18 *16S rRNA* sequences in total.

The thirteen newly obtained *COI* sequences were collapsed in six haplotypes deposited in GenBank with accession numbers OQ891056–OQ891061. Two of them made an independent cluster corresponding to *Neolebiasunifasciatus* (Fig. 1). Genetic distance (p-distance) was 0.002 between haplotypes. Two more cluster together with a sequence of *Nannaethiopsbleheri* deposited earlier by Arroyave et al. (2013) (p-d 0.002–0.003). The remaining two new haplotypes formed an independent cluster recognized by us as *Nannaethiops* sp. that is a sister to *Nannaethiopsbleheri* (Fig. 1). In general, haplotypes of the genera *Nannoethiops* and *Neolebias* comprise a monophyletic group without a clear division into two genera (Fig. 1). This is fully consistent with the conclusion of Géry and Zarske (2003) – supported by Arroyave et al. (2013) and Lavoué et al. (2017) – who considered *Neolebias* as a junior synonym of *Nannaethiops*.

The seven newly obtained *16S rRNA* sequences were collapsed in three haplotypes. One of them appeared to be identical to the sequence (JX985103) earlier deposited in GenBank for *Neolebiasunifasciatus* by Lavoue et al. (2017). Two other haplotypes we deposited in GenBank with the accession numbers OQ911366 and OQ911367. The former cluster together with the haplotype deposited for *Nannaethiopsbleheri* by Lavoué et al. (2017) (p-d 0.003); the latter belongs to the *Nannaethiops* sp. clade.

In summary, both the *COI* and *16S rRNA* analyses support: (1) our identification of *Nannaethiopsbleheri*; (2) the distinctiviness of *Nannaethiops* sp.; and (3) the *16S rRNA* analysis supports our identification of *Neolebiasunifasciatus*.

### ﻿Cytogenetic analysis

**Figure 2.**
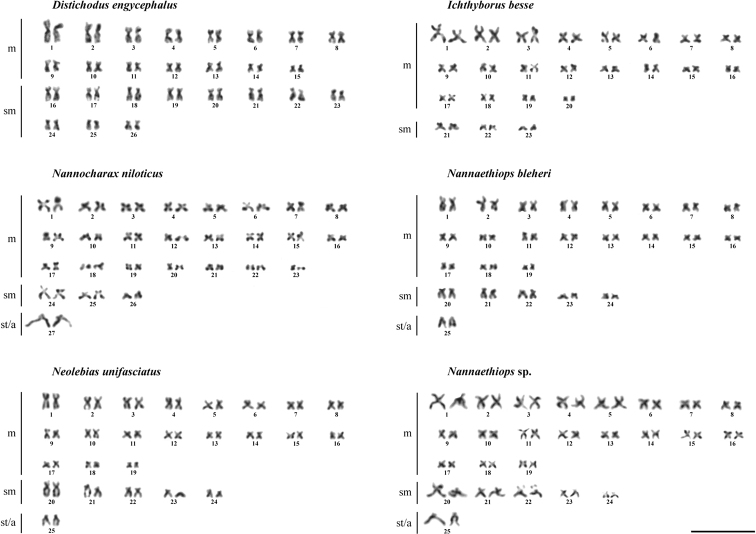
Karyotypes of six representatives of the family Distichodontidae. Scale bar: 10 μm.

The karyotype of *Distichodusengycephalus* has 2n = 52 and consists of 30 metacentrics and 22 submetacentrics, FN = 104 (Fig. 2). It differs substantially from the karyotype of *D.affinis* (2n = 48, 32m + 16sm, FN = 96) reported by Rab et al. (1998) (Table 2). No distinguishable sex chromosomes were observed in complements of *D.engycephalus*, similar to the finding by Rab et al. (1998) in *D.affinis*. This is true for all distichodontids studied by us.

**Table 2.** Cytogenetically studied taxa of the order Cithariniformes. Diploid chromosome number (2n), karyotypic formula, fundamental number (FN) and geographic origin.

Taxon	2n	Karyotypic formula	FN	Origin	References
**Family Citharinidae**					
*Citharinuscitharus* (Geoffroy St. Hilaire, 1809)	40	26m + 14sm	80	West Africa (fish store)	Simanovsky et al. 2022
*Citharinuslatus* Muller et Troschel, 1844	44	30m + 14sm	88	White Nile Basin, southwest Ethiopia	Simanovsky et al. 2022
**Family Distichodontidae**					
*Distichodusaffinis* Günther, 1873	48	32m + 16sm	96	Unknown (aquarium stock)	Rab et al. 1998
*Distichodusengycephalus* Günther, 1864	52	30m + 22sm	104	White Nile Basin, southwest Ethiopia	This study
*Ichthyborusbesse* (Joannis, 1835)	46	40m + 6sm	92	White Nile Basin, southwest Ethiopia	This study
*Nannocharaxniloticus* (Joannis, 1835)	54	46m + 6sm + 2st/a	106	White Nile Basin, southwest Ethiopia	This study
*Nannaethiopsbleheri* Géry et Zarske, 2003	50	38m + 10sm + 2st/a	98	White Nile Basin, southwest Ethiopia	This study
*Nannaethiops* sp.	50	38m + 10sm + 2st/a	98	West Africa (fish store)	This study
*Neolebiasunifasciatus* Steindachner, 1894	50	38m + 10sm + 2st/a	98	West Africa (fish store)	This study

The karyotype of *Ichthyborusbesse* has 2n = 46 and consists of 40 metacentrics and 6 submetacentrics, FN = 92. The karyotype of *Nannocharaxniloticus* has 2n = 54 and consists of 46 metacentrics, 6 submetacentrics, and 2 subtelocentrics/acrocentrics, FN = 106. The latter species exhibits the highest numbers of chromosomes and chromosome arms among all distichodontids studied (Table 2).

The karyotypes of *Nannaethiopsbleheri*, *Nannaethiops* sp. and *Neolebiasunifasciatus* appeared to be similar. These karyotypes have 2n = 50 and consists of 38 metacentric, 10 submetacentric, and 2 subtelocentrics/acrocentrics, FN = 96. These taxa, along with *Nannocharaxniloticus*, possess the only pair of monoarmed chromosomes; the other distichodontids studied have exclusively biarmed chromosomes in their compliments.

The molecular phylogeny of the order Cithariniformes as it is reconstructed by Arroyave et al. (2013) and Lavoué et al. (2017) is as follows. The family Citharinidae is a sister group to the family Distichodontidae. *Xenocharax* Günther, 1867 comprises a sister group to all other distichodontids. *Nannaethiops* + *Neolebias* represent a sister group to other distichodontids excluding *Xenocharax*. *Monostichodus* Vaillant in Rivière, 1886 + *Ichthyborus* comprise a sister group to all remaining distichodontids. Branching of the remaining three clades (*Distichodus + Paradistichodus* Pellegrin, 1922, *Nannocharax*, *Belonophago* Giltay, 1929 + *Phago* Günther, 1865 with the related genera) is not well supported and different in Arroyave et al. (2013) and Lavoué et al. (2017). Nevertheless the monophyly of the each of three groups is well supported. Thus, we analysed the representatives of four clades out of six excluding *Xenocharax* and *Belonophago* + *Phago* with the related genera.

There is an apparent correspondence between molecular phylogeneetic and cytogenetic data. There are differences in cytogenetic characteristics between *Distichodus* (2n = 48–52), *Ichthyborus* (2n = 46), *Nannocharax* (2n = 54) and *Nannaethiops* + *Neolebias* (2n = 50) representing the four different clades revealed by phylogenetic analyses. Moreover, there are differences in cytogenetic characteristics between all these distichodontids and the two species of *Citharinus* (2n = 40–44) (Table 2). These data clearly suggest a substantial role of chromosome fusions/fissions in the evolution of Cithariniformes karyotypes.

Regarding variation within the clades, we see two opposing trends. Two species of *Distichodus*, *D.affinis* and *D.engycephalus*, differ both in diploid chomosome numbers and karyotypic formulae. On the contrary, no differences were found between karyotypes of *Nannaethiopsbleheri*, *Nannaethiops* sp. and *Neolebiasunifasciatus* representing another clade. The latter point corroborates the position of authors who considered *Neolebias* as a junior synonym of *Nannaethiops* (Géry and Zarske 2003, Arroyave et al. 2013, Lavoué et al. 2017). Variability of karyotype structure in the genus *Distichodus* makes it possible to use the cytogenetic data in its taxonomy when a sufficient array of such data is accumulated. The same is true for the family Distichodontidae as a whole.

Due to the lack of data on the diversity of karyotypes in both the families Citharinidae and Distichodontidae it might be premature to make assumptions about the trend of karyotype evolution in the order Cithariniformes. The great prevalence of biarmed chromosomes (the karyotypes of most species contain exclusively biarmed chromosomes) is a distinctive characteristic of Cithariniformes compared to Characiformes and Siluriformes, sister groups to Cithariniformes. Characiformes and Siluriformes are characterized by karyotypes with various proportions of biarmed and monoarmed chromosomes (Arai 2011; Simanovsky et al. 2022). There is reason to suggest that the ancestral karyotype of Cithariniformes consisted exclusively/predominantly of biarmed chromosomes. However, the karyotypes of representatives of the basal group of Distichodontidae – genus *Xenocharax* – have yet to be determined. Thus, the cytogenetic information about this genus and other unexamined taxa of Cithariniformes would be of great interest.

## Supplementary materials

Supplementary material 1 Supporting information Data type pdf Explanation note supplementary text, tables S1–S3, figures S1–S4, supplementary references. https://binary.pensoft.net/file/929945This dataset is made available under the Open Database License (http://opendatacommons.org/licenses/odbl/1.0/). The Open Database License (ODbL) is a license agreement intended to allow users to freely share, modify, and use this Dataset while maintaining this same freedom for others, provided that the original source and author(s) are credited.Sergey A. Simanovsky, Dmitry A. Medvedev, Fekadu Tefera, Alexander S. Golubtsov

## References

[B1] Arai R (2011) Fish karyotypes – a Check List. Springer, 340 pp. 10.1007/978-4-431-53877-6

[B2] Arroyave J Denton JSS Stiassny MLJ (2013) Are characiform fishes Gondwanan in origin? Insights from a time-scaled molecular phylogeny of the Citharinoidei (Ostariophysi: Characiformes). PLoS ONE 8(10): e77269. 10.1371/journal.pone.0077269 PMC3792904 24116219

[B3] Bertollo LAC Cioffi MB Moreira-Filho O (2015) Direct chromosome preparation from freshwater teleost fishes. In: Ozouf-Costaz C, Pisano E, Foresti F, de Almeida Toledo LF (Eds) Fish Cytogenetic Techniques: Ray-Fin Fishes and Chondrichthyans, CRC Press, Boca Raton, 21–26. 10.1201/b18534

[B4] Calcagnotto D Schaefer SA DeSalle R (2005) Relationships among characiform fishes inferred from analysis of nuclear and mitochondrial gene sequences. Molecular Phylogenetics and Evolution 36(1): 135–153. 10.1016/j.ympev.2005.01.004 15904862

[B5] Decru E Moelants T De Gelas K Vreven E Verheyen E Snoeks J (2016) Taxonomic challenges in freshwater fishes: a mismatch between morphology and DNA barcoding in fish of the north-eastern part of the Congo basin. Molecular Ecology Resources 16: 342–352. 10.1111/1755-0998.12445 26186077

[B6] Dornburg A Near TJ (2021) The emerging phylogenetic perspective on the evolution of Actinopterygian fishes. Annual Review of Ecology, Evolution, and Systematics 52(1): 427–452. 10.1146/annurev-ecolsys-122120-122554

[B7] Edwards U Rogall T Blöcker H Emde M Böttger EC (1989) Isolation and direct complete nucleotide determination of entire genes: characterization of a gene coding for 16S ribosomal RNA. Nucleic Acids Research 17: 7843–7853. 10.1093/nar/17.19.7843 2798131 PMC334891

[B8] Eschmeyer WN Fricke R van der Laan R (2023) Catalog of Fishes: Genera, Species, References. http://researcharchive.calacademy.org/research/ichthyology/catalog/fishcatmain.asp [accesed 09.06.2023]

[B9] Felsenstein J (1985) Confidence limits on phylogenies: An approach using the bootstrap. Evolution 39: 783–791. 10.1111/j.1558-5646.1985.tb00420.x 28561359

[B10] Froese R Pauly D (2023) FishBase. http://www.fishbase.org [accessed 01.08.2023]

[B11] Géry J Zarske A (2003) *Nannaethiopsbleheri* sp. n. – ein neuer, afrikanischer Salmler (Teleostei, Characiformes, Distichodidae) vom oberen Weißen Nil in Südwestäthiopien. Zoologische Abhandlungen (Dresden) 53: 37–45.

[B12] Golubtsov AS Darkov AA Dgebuadze YY Mina MV (1995) An artificial key to fish species of the Gambela region (the White Nile basin in the limits of Ethiopia). Joint Ethio-Russian Biological Expedition, Addis Abeba, 84 pp.

[B13] Gosse J-P Coenen EJ (1990) Distichodontidae. In: Lévêque C Paugy D Teugels GG (Eds) Faune des poissons d’eaux douces et saumâtres de l’Afrique de l’Ouest, T.1, Paris, ORSTOM; Tervuren, MRAC, 237–260.

[B14] Hasegawa M Kishino H Yano T (1985) Dating the human-ape split by a molecular clock of mitochondrial DNA. Journal of Molecular Evolution 22: 160–174. 10.1007/BF02101694 3934395

[B15] Huelsenbeck JP Ronquist F (2001) MRBAYES: Bayesian inference of phylogenetic trees. Bioinformatics 17: 754–755. 10.1093/bioinformatics/17.8.754 11524383

[B16] Kligerman AD Bloom SE (1977) Rapid chromosome preparations from solid tissues of fishes. Journal of the Fisheries Research Board of Canada 34(2): 266–269. 10.1139/f77-039

[B17] Kumar S Stecher G Li M Knyaz C Tamura K (2018) MEGA X: Molecular Evolutionary Genetics Analysis across computing platforms. Molecular Biology and Evolution 35: 1547–1549. 10.1093/molbev/msy096 29722887 PMC5967553

[B18] Melo BF Sidlauskas BL Near TJ Roxo FF Ghezelayagh A Ochoa LE Stiassny MLJ Arroyave J Chang J Faircloth BC Macguigan DJ Harrington RC Benine RC Burns MD Hoekzema K Sanches NC Maldonado-Ocampo JA Castro RMC Foresti F Alfaro ME Oliveira C (2022) Accelerated diversification explains the exceptional species richness of tropical characoid fishes. Systematic Biology 71(1): 78–92. 10.1093/sysbio/syab040 PMC9034337 34097063

[B19] Lavoué S Arnegard ME Rabosky DL McIntyre PB Arcila D Vari RP Nishida M (2017) Trophic evolution in African citharinoid fishes (Teleostei: Characiformes) and the origin of intraordinal pterygophagy. Molecular Phylogenetics and Evolution 113: 23–32. 10.1016/j.ympev.2017.05.001 28478196

[B20] Levan A Fredga K Sandberg A (1964) Nomenclature for centromeric position on chromo- somes. Hereditas 52: 201–220. 10.1111/j.1601-5223.1964.tb01953.x

[B21] Nei M Kumar S (2000) Molecular evolution and phylogenetics. Oxford University Press, 348 pp.

[B22] Nelson JS Grande T Wilson MVH (2016) Fishes of the World, Fifth Edition, John Wiley & Sons, Inc., Hoboken, New Jersey, 707 pp. 10.1002/9781119174844

[B23] Posada D (2008) jModelTest: phylogenetic model averaging. Molecular Biology and Evolution 25: 1253–1256. 10.1093/molbev/msn083 18397919

[B24] Rab P Rabova M Ozouf-Costaz C (1998) Karyotype analysis of the African citharinid fish *Distichodusaffinis* (Osteichthyes, Characiformes) by different staining techniques. Journal of African Zooljgy 112: 185–191.

[B25] Ronquist F Huelsenbeck JP (2003) MrBayes 3: Bayesian phylogenetic inference under mixed models. Bioinformatics 19: 1572–1574. 10.1093/bioinformatics/btg180 12912839

[B26] Simanovsky SA Medvedev DA Tefera F Golubtsov AS (2022) First cytogenetic data on Afrotropical lutefishes (Citharinidae) in the light of karyotype evolution in Characiformes. Comparative Cytogenetics 16(2): 143–150. 10.3897/compcytogen.v16.i2.79133 36761810 PMC9849050

[B27] Sonet G Snoeks J Nagy ZT Vreven E Boden G Breman FC Decru E Hanssens M Zamba AI Jordaens K Mamonekene V Musschoot T Van Houdt J Van Steenberge M Wamuini SL Verheyen E (2019) DNA barcoding fishes from the Congo and the Lower Guinean provinces: Assembling a reference library for poorly inventoried fauna. Molecular Ecology Resources 19: 728–743. 10.1111/1755-0998.12983 30576073

[B28] Stackebrandt E Liesack W (1993) Nucleic acids and classification. In: Goodfellow M O’Donnell AG (Eds) Handbook of new bacterial systematics.London, Academic Press, 152–189.

[B29] Tamura K Nei M (1993) Estimation of the number of nucleotide substitutions in the control region of mitochondrial DNA in humans and chimpanzees. Molecular Biology and Evolution 10: 512–526. 10.1093/oxfordjournals.molbev.a040023 8336541

[B30] Ward RD Zemlak TS Innes BH Last PR Hebert PD (2005) DNA barcoding of Australia’s fish species. Philosophical Transactions of the Royal Society B: Biological sciences 360: 1847–1857. 10.1098/rstb.2005.1716 PMC1609232 16214743

