# Supplementary material for: ﻿Divergent karyotypes in five genera of the African endemic fish family Distichodontidae (Cithariniformes, Osteichthyes)
Source: Comp Cytogenet. 2023 Nov 2;17:251–62. doi: 10.3897/compcytogen.17.107744 (PMC10636602; doi:10.3897/compcytogen.17.107744)
Supplement: Supplementary material 1 — Supporting information [file comparative_cytogenetics-17--251_article-107744__-s001.pdf]

Supporting information for

Divergent karyotypes in five genera of the African endemic fish family Distichodontidae (Citholariniformes, Osteichthyes)

Sergey A. Simanovsky, Dmitry A. Medvedev, Fekadu Tefera, Alexander S. Golubtsov

Corresponding author: *Sergey A. Simanovsky* (sergey.a.simanovsky@gmail.com)

Supporting information includes:

Supplementary Text 1

Supplementary Table 1 (Table S1)

Supplementary Table 2 (Table S2)

Supplementary Table 3 (Table S3)

Supplementary Figure 1 (Figure S1)

Supplementary Figure 2 (Figure S2)

Supplementary Figure 3 (Figure S3)

Supplementary Figure 4 (Figure S4)

Supplementary References

Supplementary Text 1

Within the studied *COI* sequences of 615 bp there were 223 variable sites including 197 that were parsimonially informative. Within the studied *16S rRNA* sequences of 387 bp there were 80 variable sites including 48 that were parsimonially informative. Complete information about all sequences involved in the molecular phylogenetic analyses is given in Table S1. Genetic distances between *COI* haplotypes are given in Table S2. Genetic distances between *16S rRNA* haplotypes are given in Table S3. Phylogenetic trees constructed using maximum parsimony (MP) (Nei and Kumar 2000) and Bayesian inference (BI) methods for *COI* and *16S rRNA* are presented in Fig. S1-S4.

Table S1. Sequence data involved in the molecular phylogenetic analysis. Species names are given in accordance with the GenBank NCBI database.

Taxon	Haplotype (GenBank Accession number)		Origin	Coordinates	Voucher	References
	COI	16S				
Belonophago						
Belonophago hutsebouti	KF541759		Democratic Republic of the Congo, Salonga National Park, Nkombe-Dunda, Luilaka River, downstream of village, near confluence of Luilaka and Lila Rivers.	2°40'17.3"S 21°43'15.5"E	AMNH 241850	Arroyave et al. 2013
	KF541760		Democratic Republic of the Congo, Salonga National Park, Nkema Asondzi, Luilaka River		AMNH 241851	Arroyave et al. 2013
	HM418229		Democratic Republic of the Congo: Equateur.	2°13'27.7"S 21°11'06.4"E		Unpublished
		JX985128	Republic of the Congo: Congo River basin, Odzala			Lavoué et al. 2017
Citharinus						
Citharinus citharus	KF541750		Benin, Niger river at tunga village		AMNH 226441	Arroyave et al. 2013
	KF541749		Benin, Niger river at tunga village		AMNH 226441	Arroyave et al. 2013
	HM882704		Nigeria: Abai, South-East, Ebonyi, Afikpo			Nwani et al. 2011
	HM882705		Nigeria: Anambra, South-East, Otuocha			Nwani et al. 2011
	HM882710		Nigeria: Abai, South-East, Ebonyi, Afikpo			Nwani et al. 2011
	HM882713		Nigeria: Anambra, South-East, Otuocha			Nwani et al. 2011
	HM882715		Nigeria: Anambra, South-East, Otuocha			Nwani et al. 2011
	JF510508		Nigeria: Abai, South-East, Ebonyi, Afikpo.	5°54'36.0"N 8°03'21.6"E		Nwani et al. 2011
	JF510509		Nigeria: Abai, South-East, Ebonyi, Afikpo.	5°54'36.0"N 8°03'21.6"E		Nwani et al. 2011

		AY787989	Benin, Niger river at tunga village		AMNH 226441	Calcagnotto et al. 2005
Distichodus						
Distichodus nefasch	MT300595		Ethiopia, Southern Nations, Nationalities and Peoples, Omo River.	4°47'33.9"N 36°02'22.4"E	AMNH 264420	Arroyave et al. 2020
Distichodus sexfasciatus	AB070242	AB070242				Nakatani et al. 2011
Nannaethiops						
Nannaethiops bleheri	KF541848				CU 94489	Arroyave et al. 2013
	OQ891056		Ethiopia, Interfluve of the Alvero and Gilo rivers	7°45'18.4"N 34°15'38.3"E		This study
	OQ891057		Ethiopia, Interfluve of the Alvero and Gilo rivers	7°45'18.4"N 34°15'38.3"E		This study
		JX985104	Ethiopia: Nile River basin, Gambela			Lavoué et al. 2017
		OQ911366	Ethiopia, Baziel Kebele	8°18'51.2"N 34°04'24.9"E		This study
Nannaethiops gracilis	KF541851		Democratic Republic of the Congo, Luilaka River, Salonga National Park, Luilaka River, Boangi village		AMNH 241888	Arroyave et al. 2013
	KF541852		Democratic Republic of the Congo, Luilaka River, Salonga National Park, Luilaka River, Boangi village		AMNH 241888	Arroyave et al. 2013
Nannaethiops unitaeniatus	KF541849		Cameroon, South Province, Region of Kribi, stream near village of Massa'a		AMNH 249555	Arroyave et al. 2013
	KF541850		Cameroon, South Province, Region of Kribi, stream near village of Massa'a		AMNH 249555	Arroyave et al. 2013
		JX985105	Gabon: Rabi			Lavoué et al. 2017
Nannaethiops sp	OQ891061	OQ911367	West Africa, Fish Import			This study
	OQ891060		West Africa, Fish Import			This study
Neolebias						
Neolebias ansorgii	KF541859		Democratic Republic of the Congo, Small coast stream		AMNH 251003	Arroyave et al. 2013

	KF541860				CU 93512	Arroyave et al. 2013
	KF541858		Democratic Republic of the Congo, small stream flowing into Atlantic Ocean near Moanda		AMNH 250442	Arroyave et al. 2013
	HM418212		Democratic Republic of the Congo: Bas-Congo, Aquarium Trade			Sonet et al. 2019
	HM418213		Democratic Republic of the Congo: Bas-Congo, Aquarium Trade			Sonet et al. 2019
		AY788058				Calcagnotto et al. 2005
		JX985107	Gabon: Doumvou			Lavoué et al. 2017
Neolebias powelli		AY788061	Nigeria, Niger delta		AMNH 233408	Calcagnotto et al. 2005
Neolebias trewavasae	KF541857				AMNH uncataloged	Arroyave et al. 2013
	KF541853		Cameroon, South Province, Stream near Metondo Village, Kribia-Ebolowa Road		AMNH 249536	Arroyave et al. 2013
		JX985132	Gabon: Ivindo River, Loa Loa			Lavoué et al. 2017
Neolebias trilineatus	KF541856				CU 91513	Arroyave et al. 2013
	KF541854		Democratic Republic of the Congo, Salonga National Park, Nkombe-Dunda, Luilaka River, downstream of village, near confluence of Luilaka and Lila Rivers.	2°40'17.3"S 21°43'15.5"E	AMNH 247928	Arroyave et al. 2013
	KF541855		Democratic Republic of the Congo, Salonga National Park, Nkombe-Dunda, Luilaka River, downstream of village, near confluence of Luilaka and Lila Rivers.	2°40'17.3"S 21°43'15.5"E	AMNH 247928	Arroyave et al. 2013
	MK074510		Republic of the Congo: Congo, Lefini, Marigotsn tout pres du confluent.	2°59'24.0"S 15°30'00.0"E		Sonet et al. 2019
	MK074511		Republic of the Congo: Congo, Lefini, Marigotsn tout pres du confluent.	2°59'24.0"S 15°30'00.0"E		Sonet et al. 2019
	KT193336		Democratic Republic of the Congo, Itimbiri River			Decru et al. 2016
	HM418214		Democratic Republic of the Congo: Equateur.	2°40'17.3"S 21°43'15.6"E		Sonet et al. 2019

	HM418215		Democratic Republic of the Congo: Equateur.	2°40'17.3"S 21°43'15.6"E		Sonet et al. 2019
		AY788063	Africa Fish Import		AMNH233439	Calcagnotto et al. 2005
Neolebias unifasciatus		JX985103	Benin: Oueme River			Lavoué et al. 2017
	OQ891059	JX985103	West Africa, Fish Import			This study
	OQ891058	JX985103	West Africa, Fish Import			This study

Table S2. Estimates of genetic distances (p-distance) between haplotypes *COI* (615 bp).

Species	GenBank NCBI		1	2	3	4	5	6	7	8	9	10	11	12	13	14	15	16	17	18	19	20	21	22	23
Neolebias unifasciatus	OQ891058	1																							
	OQ891059	2	0,002																						
Nannaethiops bleheri	KF541848	3	0,112	0,111																					
	OQ891056	4	0,111	0,109	0,002																				
	OQ891057	5	0,114	0,112	0,002	0,003																			
Nannaethiops sp.	OQ891060	6	0,117	0,115	0,037	0,036	0,039																		
	OQ891061	7	0,114	0,112	0,052	0,050	0,054	0,031																	
Nannaethiops unitaeniatus	KF541850	8	0,107	0,106	0,055	0,054	0,057	0,037	0,046																
Neolebias trilineatus	KF541856	9	0,104	0,106	0,117	0,115	0,119	0,119	0,111	0,115															
	MK074510	10	0,114	0,115	0,114	0,112	0,112	0,107	0,102	0,109	0,054														
	KT193336	11	0,102	0,104	0,111	0,109	0,112	0,111	0,102	0,102	0,059	0,062													
	HM418214	12	0,104	0,106	0,115	0,114	0,117	0,111	0,107	0,111	0,063	0,067	0,031												
	HM418215	13	0,107	0,109	0,115	0,114	0,117	0,111	0,107	0,109	0,063	0,067	0,031	0,003											
Nannaethiops gracilis	KF541851	14	0,120	0,119	0,130	0,128	0,132	0,128	0,120	0,125	0,102	0,101	0,099	0,111	0,111										
	KF541852	15	0,119	0,117	0,132	0,130	0,133	0,130	0,122	0,127	0,104	0,102	0,101	0,112	0,112	0,002									
Neolebias trewavasae	KF541857	16	0,151	0,150	0,145	0,143	0,146	0,141	0,138	0,138	0,143	0,137	0,137	0,128	0,130	0,148	0,146								
	KF541853	17	0,141	0,140	0,145	0,143	0,146	0,146	0,135	0,140	0,137	0,137	0,133	0,132	0,133	0,143	0,141	0,036							
Neolebias ansorgii	KF541859	18	0,164	0,163	0,187	0,185	0,187	0,182	0,187	0,179	0,176	0,180	0,182	0,167	0,169	0,179	0,179	0,148	0,150						
Belonophago hutsebouti	HM418229	19	0,174	0,172	0,190	0,189	0,189	0,192	0,189	0,189	0,180	0,177	0,171	0,163	0,163	0,190	0,192	0,174	0,177	0,195					
Distichodus nefasch	MT300595	20	0,148	0,146	0,163	0,161	0,161	0,164	0,164	0,161	0,159	0,163	0,167	0,156	0,158	0,171	0,172	0,150	0,148	0,161	0,137				
Distichodus sexfasciatus	AB070242	21	0,146	0,145	0,169	0,167	0,171	0,166	0,166	0,161	0,154	0,169	0,154	0,159	0,158	0,161	0,163	0,169	0,171	0,169	0,143	0,089			
Citharinus citharus	HM882704	22	0,195	0,193	0,179	0,177	0,177	0,190	0,189	0,184	0,195	0,193	0,195	0,198	0,200	0,193	0,195	0,205	0,200	0,195	0,193	0,195	0,180		
	HM882705	23	0,203	0,202	0,177	0,176	0,179	0,187	0,185	0,184	0,179	0,190	0,184	0,184	0,187	0,202	0,203	0,193	0,192	0,207	0,198	0,189	0,192	0,109	

Table S3. Estimates of genetic distances (p-distance) between *16S r RNA* haplotypes (387 bp)

Species	GenBank NCBI		1	2	3	4	5	6	7	8	9	10	11	12
Neolebias unifasciatus	JX985103	1												
Nannaethiops bleheri	JX985104	2	0,039											
	OQ911366	3	0,039	0,003										
Nannaethiops sp.	OQ911367	4	0,034	0,016	0,018									
Nannaethiops unitaeniatus	JX985105	5	0,039	0,023	0,023	0,018								
Neolebias trilineatus	AY788063	6	0,036	0,029	0,029	0,029	0,034							
Neolebias trewavasae	JX985132	7	0,076	0,070	0,068	0,070	0,073	0,055						
Neolebias ansorgii	JX985107	8	0,084	0,076	0,073	0,079	0,076	0,073	0,084					
Neolebias powelli	AY788061	9	0,103	0,105	0,103	0,097	0,100	0,105	0,111	0,079				
Belonophago hutsebouti	JX985128	10	0,096	0,094	0,091	0,086	0,089	0,096	0,117	0,100	0,105			
Distichodus sexfasciatus	AB070242	11	0,076	0,086	0,086	0,076	0,078	0,068	0,073	0,073	0,092	0,089		
Citharinus citharus	AY787989	12	0,110	0,118	0,118	0,110	0,115	0,107	0,102	0,113	0,122	0,131	0,097	

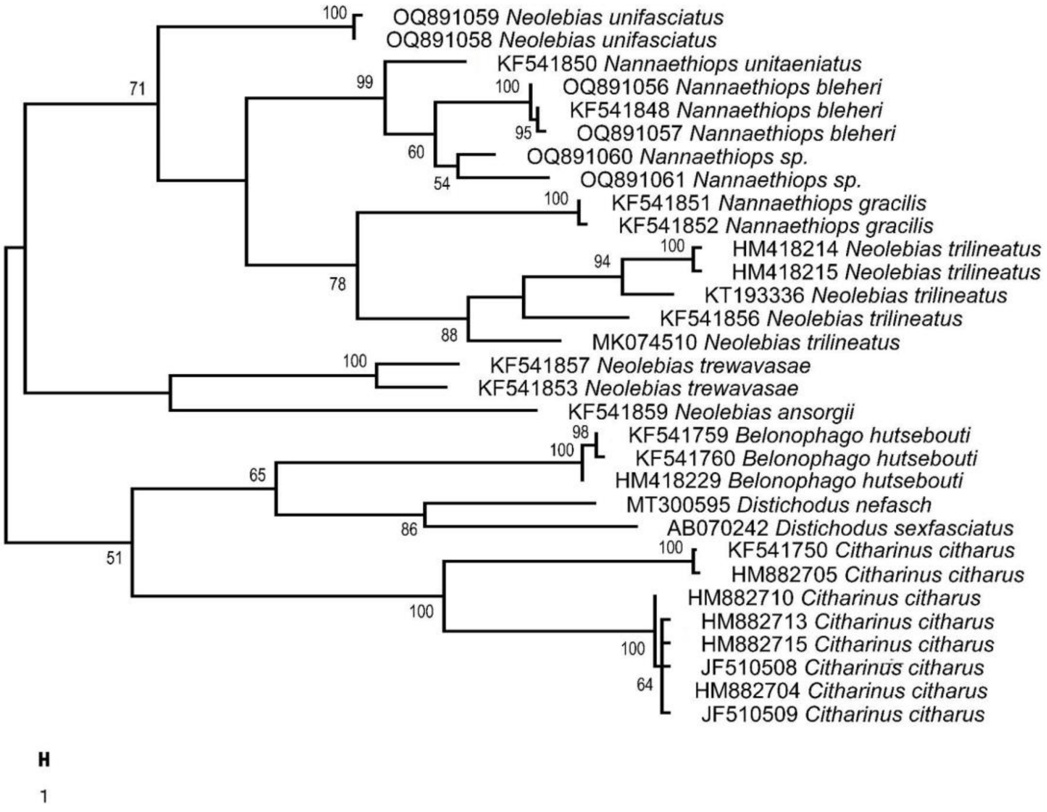

Fig. S1. Maximum Parsimony (MP) trees with compressed subtrees based on 615-bp *COI* fragment. Length of branches is proportional to the genetic distances between haplotypes; bootstrap support (Felsenstein, 1985) is indicated next to the branching nodes and calculated from 1000 replicas (not specified - bootstrap support is less than 50%).

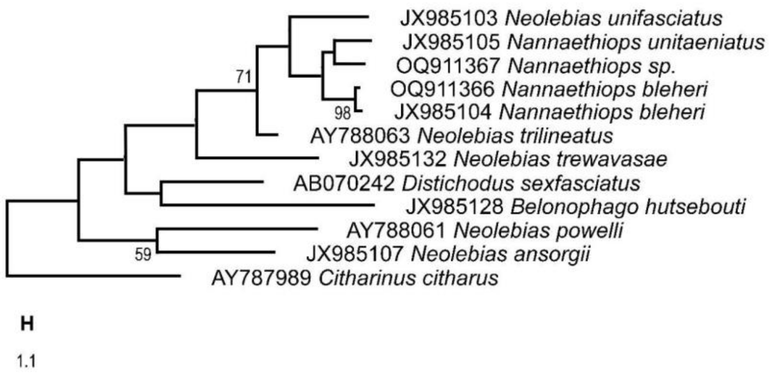

Fig. S2. Maximum Parsimony (MP) trees with compressed subtrees based on 387-bp *16S rRNA* fragment. Length of branches is proportional to the genetic distances between haplotypes; bootstrap support (Felsenstein 1985) is indicated next to the branching nodes and calculated from 1000 replicas (not specified - bootstrap support is less than 50%).

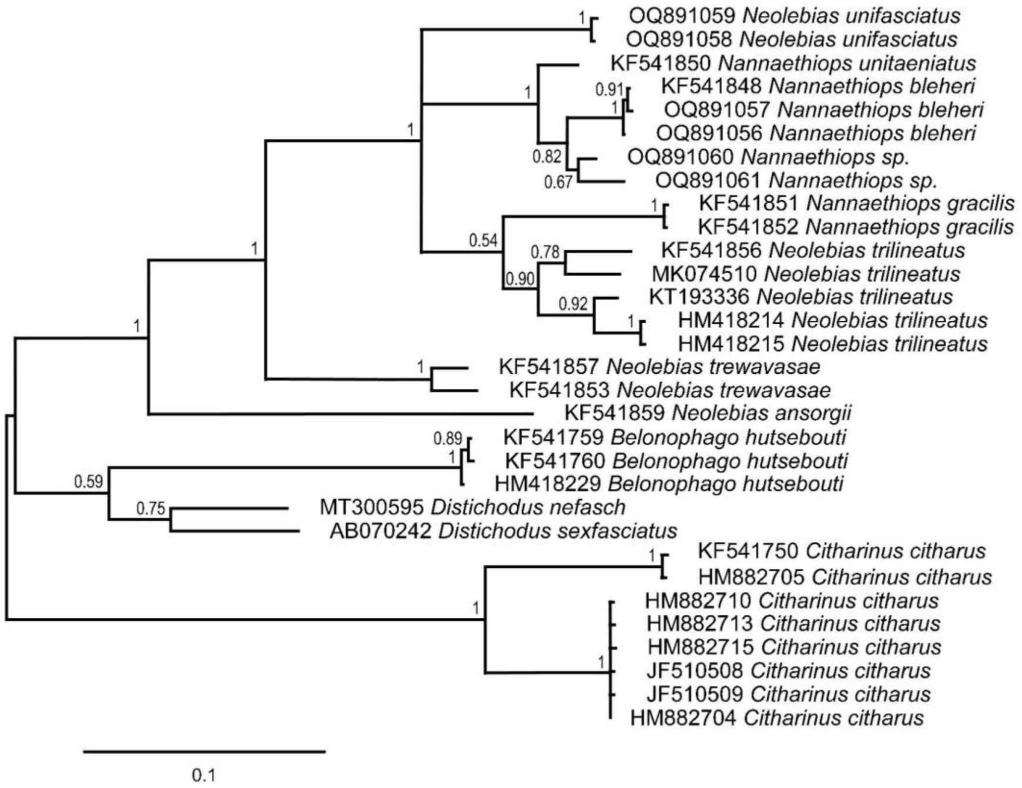

Fig. S3. Bayesian Inference (BI) trees with compressed subtrees based on 615-bp *COI* fragment. Length of branches is proportional to the genetic distances between haplotypes; support is indicated next to the branching nodes and calculated from 10000 replicas (not specified - bootstrap support is less than 0.5).

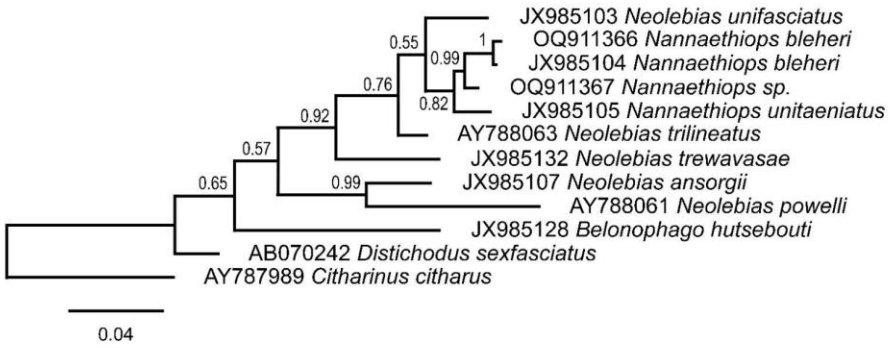

Fig. S4. Bayesian Inference (BI) trees with compressed subtrees based on 387-bp *16S rRNA* fragment. Length of branches is proportional to the genetic distances between haplotypes; support is indicated next to the branching nodes and calculated from 10000 replicas (not specified - bootstrap support is less than 0.5).

Supplementary References

Arroyave J, Denton JSS, Stiassny MLJ (2013) Are characiform fishes Gondwanan in origin? Insights from a time-scaled molecular phylogeny of the Citharinoidei (Ostariophysi: Characiformes). PLoS ONE 8(10): e77269. https://doi.org/10.1371/journal.pone.0077269

Arroyave J, Denton JSS, Stiassny MLJ (2020) Pattern and timing of diversification in the African freshwater fish genus Distichodus (Characiformes: Distichodontidae). BMC Evol Biol. 20(1): 48. https://doi.org/10.1186/s12862-020-01615-6

Calcagnotto D, Schaefer SA, DeSalle R (2005) Relationships among characiform fishes inferred from analysis of nuclear and mitochondrial gene sequences. Molecular Phylogenetics and Evolution 36 (1): 135–153. https://doi.org/10.1016/j.ympev.2005.01.004

Decru E, Moelants T, De Gelas K, Vreven E, Verheyen E, Snoeks J (2016) Taxonomic challenges in freshwater fishes: a mismatch between morphology and DNA barcoding in fish of the north-eastern part of the Congo basin. Molecular Ecology Resources 16: 342–352. https://doi.org/10.1111/1755-0998.12445

Felsenstein J (1985) Confidence limits on phylogenies: An approach using the bootstrap. Evolution 39: 783-791. https://doi.org/10.1111/j.1558-5646.1985.tb00420.x

Lavoué S, Arnegard ME, Rabosky DL, McIntyre PB, Arcila D, Vari RP, Nishida M (2017) Trophic evolution in African citharinoid fishes (Teleostei: Characiformes) and the origin of intraordinal pterygophagy. Molecular Phylogenetics and Evolution 113: 23–32. https://doi.org/10.1016/j.ympev.2017.05.001

Nakatani M, Miya M, Mabuchi K, Saitoh K, Nishida M (2011) Evolutionary history of Otophysi (Teleostei), a major clade of the modern freshwater fishes: Pangaean origin and Mesozoic radiation. BMC Evolutionary Biology 11, 177. http://www.biomedcentral.com/1471-2148/11/177

Nei M, Kumar S (2000) Molecular evolution and phylogenetics. Oxford University Press, 348 pp.

Nwani CD, Becker S, Braid HE, Ude EF, Okogwu OI, Hanner R (2011) DNA barcoding discriminates freshwater fishes from southeastern Nigeria and provides river system-level phylogeographic resolution within some species. Mitochondrial DNA 22: 43–51. https://doi.org/10.3109/19401736.2010.536537

Sonet G, Snoeks J, Nagy ZT, Vreven E, Boden G, Breman FC, Decru E, Hanssens M, Zamba AI, Jordaens K, Mamonekene V, Musschoot T, Van Houdt J, Van Steenberge M, Wamuini SL, Verheyen E (2019) DNA barcoding fishes from the Congo and the Lower Guinean provinces: Assembling a reference library for poorly inventoried fauna. Molecular Ecology Resources 19: 728–743. https://doi.org/10.1111/1755-0998.12983
